# Low-Defect Bulk-Germanium-on-Insulator Photodetectors with Resonant Cavity Enhancement at 1550 nm for High-Resolution SWIR Imaging

**DOI:** 10.3390/nano16050316

**Published:** 2026-03-02

**Authors:** Jiale Su, Ben Li, Yuhui Ren, Junhao Du, Xiangliang Duan, Tianyu Dong, Xueyin Su, Tianchun Ye, Xuewei Zhao, Yuanhao Miao, Henry H. Radamson

**Affiliations:** 1 Research and Development Center of Optoelectronic Hybrid IC, Guangdong Greater Bay Area Institute of Integrated Circuit and System, Guangzhou 510535, China; 2 Guangzhou Noor Optoelectronic Technology Co., Ltd., Guangzhou 510530, China

**Keywords:** short-wave infrared (SWIR), bulk-GeOI, wafer bonding, photodetectors (PDs), responsivity, external quantum efficiency (EQE)

## Abstract

High-resolution short-wave infrared (SWIR) imaging requires photodetectors (PDs) with simultaneously low dark current and high responsivity. To achieve this goal, we demonstrate low-defect bulk germanium-on-insulator (bulk-GeOI) PDs designed for enhanced 1550 nm absorption and suppressed dark current via a resonant cavity and low-defect material platform. Devices were fabricated by direct bonding low-defect bulk Ge and thinning it to ~1300 nm, with an intrinsic layer thickness of only 800 nm. This design avoids epitaxial defects to lower intrinsic dark current while forming a resonant cavity for enhanced responsivity at 1550 nm. Precise doping and Al_2_O_3_/Si_3_N_4_ bilayer sidewall passivation were employed. From a design perspective, using low-defect bulk Ge minimizes the defects from epitaxial growth and reduces intrinsic dark current, while thinning the Ge layer enhances the resonant cavity effect to improve 1550 nm responsivity. Experimentally, despite the thin absorbing layer, our devices achieved nA-level dark currents (e.g., 18 nA at −1 V for 10 μm devices) alongside high responsivities. Detailed analysis indicates that this dark current is predominantly attributed to surface and sidewall defects from mesa etching, with minimal contribution from low-defect bulk material defects, validating the effectiveness of the bulk-Ge approach in suppressing intrinsic bulk leakage. Optically, the devices achieved high responsivities of 0.85 A/W (1310 nm) and 0.72 A/W (1550 nm), corresponding to external quantum efficiencies of 80.6% and 57.7%, respectively. This work establishes the bulk-GeOI platform as a promising path toward high-performance SWIR PDs, successfully decoupling high responsivity from bulk leakage and paving the way for future gains through refined surface and interface engineering.

## 1. Introduction

Short-wave infrared (SWIR) imaging, spanning approximately 1.5–3.0 μm, has emerged as a pivotal technology for applications in telecommunications [1,2,3,4,5], biomedical imaging [6,7,8], environmental monitoring, and space-based observation [9,10,11]. This spectral range is particularly advantageous because it offers reduced scattering in biological tissues, enhanced imaging contrast under low-light conditions, and selective material discrimination, which are critical for high-resolution and high-sensitivity photodetection. Current SWIR detection technologies primarily rely on InGaAs [12,13] PDs, GeSn alloys [14,15,16,17,18], colloidal quantum dot (CQD) PDs [19,20] and Ge-on-Si PDs [21,22,23,24,25], each of which presents a distinct trade-off. InGaAs devices provide well-established fabrication and high responsivity, but they are hindered by high material costs and limited compatibility with Si-based electronics. GeSn alloys allow bandgap tunability toward longer wavelengths [26,27]; however, their widespread adoption is hampered by challenges in material quality, defect control, and uniformity, which often restrict device performance and reliability. CQDs offer solution-processable fabrication and spectral tunability, yet they typically suffer from inferior carrier mobility, operational stability, and quantum efficiency compared with crystalline semiconductors [28]. Ge-on-Si PDs offer the advantages of CMOS compatibility and low cost. However, due to the 4.2% lattice mismatch between Ge and Si, the heteroepitaxial growth of Ge directly on Si substrates introduces a high density of dislocations in the Ge epilayer. These dislocations act as effective recombination centers, significantly degrading the performance of Ge-on-Si photodetectors. Collectively, these limitations underscore the pressing need for Ge-based PDs that can simultaneously achieve low dark current, high responsivity, and CMOS-compatible integration [29,30,31], satisfying the demands of next-generation high-resolution SWIR imaging systems.

GeOI technology has emerged as a promising platform for SWIR, which is fully compatible with flip-chip technology [32]. In a typical GeOI structure, a thin Ge layer is bonded onto an insulating oxide layer atop a Si substrate. This configuration mitigates substrate leakage relative to bulk Ge and provides partial compatibility with Si photonics, while avoiding lattice-mismatch issues associated with epitaxial growth [33,34,35,36]. Despite these advantages, conventional GeOI devices face two fundamental limitations. First, even with the insulating layer, dark currents typically remain at the nA level, constraining detector sensitivity under low-light or high-resolution imaging conditions. Second, the intrinsic absorption of Ge near 1550 nm is limited [37], resulting in suboptimal responsivity at this technologically critical wavelength. Therefore, a refined GeOI platform capable of simultaneously suppressing dark current and enhancing optical absorption at telecom wavelengths is essential for high-performance SWIR PDs.

To address these challenges, we propose and demonstrate a novel PD based on a low-defect bulk-GeOI platform. Our approach employs high-quality bulk Ge [38,39,40] as the starting material, which is thinned to ~1300 nm with an intrinsic layer thickness of 800 nm to form an optical cavity for resonant enhancement at 1550 nm while maintaining efficient carrier collection. Key to this platform is the use of direct wafer bonding of a bulk Ge wafer, a process that avoids the crystalline defects commonly introduced during heteroepitaxial growth [41,42,43,44], and thus provides a superior baseline for probing bulk-related dark current mechanisms. Following bonding, the Ge layer is precisely thinned, and well-defined n-type and p-type regions are formed via ion implantation and rapid thermal annealing. To tackle surface-induced leakage, an Al_2_O_3_/Si_3_N_4_ bilayer sidewall passivation [45,46] is applied after mesa etching. This integrated fabrication strategy establishes a controlled platform to dissect the contributions of bulk versus surface defects to dark current and to optimize optical absorption via cavity design. In this work, we present a comprehensive investigation of this platform. We elucidate the full implications of our design choices on electrical performance and optical responsivity, with a particular focus on the interplay between bulk material quality and surface leakage mechanisms. By concurrently addressing intrinsic material limitations and fabrication-induced parasitic effects, this work demonstrates a pathway toward CMOS-compatible SWIR PDs that concurrently achieve low dark current and high quantum efficiency, thereby advancing the development of next-generation high-resolution SWIR imaging systems.

## 2. Experimental Methods

**Figure 1:**
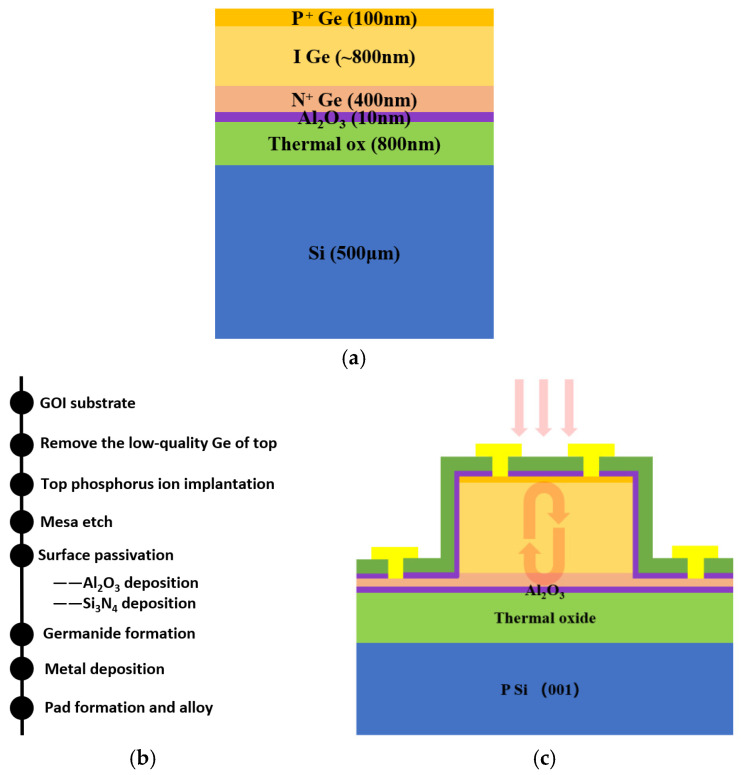
(**a**) Schematic of the bonded wafer structure. (**b**) Key process flow for device fabrication. (**c**) Cross-sectional schematic of the PIN mesa PD on the low-defect bulk-GeOI platform, the pink arrows indicate perpendicularly incident light and resonance phenomenon within the device.

An intrinsic bulk Ge wafer with an ultra-low defect density (<300 cm^−2^) was employed as the donor wafer for the fabrication of a GeOI substrate via direct wafer bonding. Prior to the bonding process, phosphorus ions were implanted into the Ge wafer at an energy of 120 keV with a dose of 1.8 × 10^15^ cm^−2^ to form an n-type layer with a thickness of approximately 400 nm [47,48]. Subsequently, a 20 nm Al_2_O_3_ layer was deposited on the Ge surface via atomic layer deposition (ALD), serving as a bonding interlayer and enhancing the interfacial quality of the final GOI wafer. The handle wafer comprised a Si (100) substrate with an 800 nm thermally grown SiO_2_ layer. Following wafer bonding, the wafer pair underwent annealing at 300 °C for 3 h in a nitrogen ambient [49]. The bonded Ge layer was then thinned to a final thickness of ~1.3 μm through mechanical grinding followed by chemical mechanical polishing (CMP). To complete the diode junction, a 100 nm p-type Ge region was formed by boron ion implantation at 30 keV with a dose of 2 × 10^15^ cm^−2^, followed by rapid thermal annealing at 500 °C for 60 s in N_2_ to activate the dopants and to prevent significant phosphorus ion redistribution during the thermal annealing process [50,51,52]. The schematic of the resulting bonded wafer structure is depicted in Figure 1a. Device fabrication was carried out on the prepared GOI platform. Mesa isolation was achieved via chlorine-based dry etching. To mitigate surface leakage, sidewall passivation was implemented by sequential deposition of a 20 nm Al_2_O_3_ layer via ALD and a 300 nm Si_3_N_4_ layer via plasma-enhanced chemical vapor deposition (PECVD), which has been shown to reduce interface state density on Ge surfaces and mitigate surface leakage. This passivation strategy is well-established to reduce interface state density on Ge surfaces, thereby improving leakage characteristics, as corroborated by previous reports [53]. For electrical contacts, ohmic contacts were formed by depositing a 10 nm Ni layer via electron-beam evaporation and annealing at 400 °C for 30 s in N_2_ to form a low-resistance NiGe alloy—a material chosen for its superior specific contact resistivity on Ge [54,55,56,57]. Finally, Ti/Al (50/300 nm) stacks were sputtered and patterned via a lift-off process to define the electrodes. The key fabrication steps and the cross-sectional structure of the final device are illustrated in Figure 1b,c, respectively.

**Figure 2:**
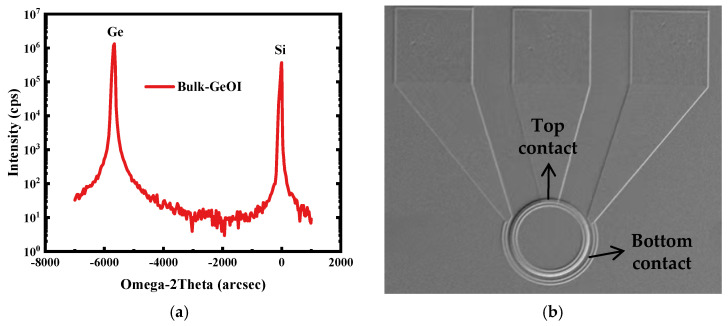
Structural characterization: (**a**) High-resolution X-ray diffraction (HRXRD) ω-2θ scan of the bulk-GeOI. (**b**) Top view of a PD in the array chip observed by SEM.

To evaluate the crystalline quality of the Ge layer in bulk GeOI wafers after the bonding and thinning process—a critical factor for carrier transport and detection efficiency in photonic devices—high-resolution X-ray diffraction (HRXRD) was employed for material analysis [58,59]. The ω-2θ scan of the (004) plane (Figure 2a) shows a sharp and symmetric Ge diffraction peak with a full width at half maximum (FWHM) of only 72 arcsec. This value is considerably narrower than the typical FWHM of heteroepitaxial Ge layers grown directly on Si substrates (>200 arcsec) [60]. From the XRD analysis, the 2θ difference between the Ge and Si peaks is measured to be 3.1454°, which is slightly larger than the theoretical value of 3.1352°, indicating a mild in-plane compressive strain (~0.053%) in the Ge layer after bonding. This slight strain is insufficient to induce defects within the material. This indicates that the bulk-Ge material retains high crystalline integrity throughout the bonding–thinning sequence without introducing significant lattice distortion or stress. These results confirm that the adopted bonding technique effectively transfers high-quality bulk Ge onto the target substrate while preserving its single-crystal character, thereby providing a crucial material foundation for fabricating high-performance optoelectronic devices. Furthermore, the surface morphology of the completed devices was examined using scanning electron microscopy (SEM), as presented in Figure 2b. A circular mesa structure was fabricated with P-type and N-type contacts on the top and bottom, respectively.

## 3. Results and Discussion

**Figure 3:**
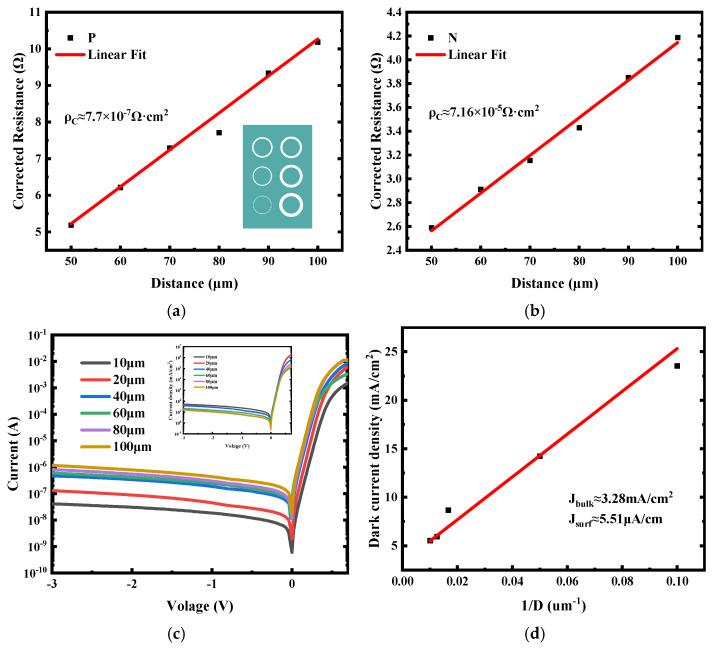
Key electrical characterizations of the fabricated bulk-GeOI PD: (**a**,**b**) Contact resistance extracted from CTLM measurements for the P-type and N-type regions, respectively, with the green inset illustrating the test layout. (**c**) Dark current as a function of reverse bias for devices with varying mesa diameters at room temperature. The illustration shows the change in dark current density with voltage. (**d**) Dark current density analysis, decomposed into contributions from bulk (J_bulk_) and surface/sidewall (J_surf_) components.

To evaluate the electrical performance of the fabricated low-defect bulk-GeOI PDs, it is essential to first assess the quality of the metal–semiconductor contacts, as contact resistance directly affects both device efficiency and dark current characteristics. The contact resistance was measured using the circular transmission line model (CTLM), which, relative to conventional TLM structures, eliminates the need for mesa etching and reduces parasitic probe resistance, thereby improving measurement reliability and accuracy [61]. As illustrated in Figure 3a,b, the measured resistance shows a clear linear dependence on the CTLM gap spacing for both N-type and P-type regions, indicating the successful formation of ohmic contacts. Linear fitting yields specific contact resistivities of 7.16 × 10^−5^ Ω·cm^2^ for the N-type region and 7.7 × 10^−7^ Ω·cm^2^ for the P-type region. These low resistivities are attributed to the formation of a low-resistance NiGe alloy during thermal annealing. The lower contact resistivity observed for the P-type region is consistent with the higher active doping concentration, which facilitates enhanced carrier tunneling at the metal–semiconductor interface. While the difference between the N-type and P-type specific contact resistivities reflects the distinct ion-implantation conditions, the precise correlation between doping profile and contact resistance in this material system warrants further investigation. With the contacts confirmed, we then examined the dark current characteristics to assess leakage behavior and overall device integrity. Figure 3c presents the dark currents for devices with different mesa diameters. As expected for area-dependent leakage, the dark current increases with mesa size. The 10 μm-diameter device exhibits 18 nA under a −1 V reverse bias, higher than typical epitaxial GeOI devices [62]. This indicates that sidewall and surface defects dominate the leakage, while bulk material defects contribute minimally. The primary contributor is likely sidewall damage induced during the chlorine-based dry-etching process used for mesa definition. Although the subsequent Al_2_O_3_/Si_3_N_4_ bilayer passivation is applied to mitigate surface leakage, it may not entirely compensate for all etching-induced defects. These observations highlight a critical opportunity for process optimization in bulk-GeOI PDs, where further refinement of etch chemistry and the introduction of post-etch treatments could substantially reduce leakage currents. Importantly, the high crystalline quality of the bonded Ge layer, confirmed by HR-XRD, indicates that bulk material defects are not the primary source of the observed dark current, reinforcing the conclusion that surface and sidewall imperfections are the dominant contributors to leakage in these devices.

In mesa-type photodiodes, the total dark current can generally be decomposed into two distinct components: the bulk dark current (J_bulk_) and the surface/sidewall dark current (J_surf_). Their combined contribution to the total dark current density, J_dark_, as a function of device diameter D, can be expressed as the following Equation (1) [63]:

(1) $$J_{\mathrm{dark}}{=J}_{\mathrm{bulk}}{+J}_{\mathrm{surf}}\times \frac{4}{D}$$

The dark current density is plotted as a function of the inverse of the mesa diameter in Figure 3d. A clear reduction in dark current density is observed with increasing device size: the 10 μm device exhibits 23.6 mA/cm^2^, whereas the 100 μm device shows a substantially lower value of 5.5 mA/cm^2^. This trend arises from the diminishing contribution of surface leakage relative to the bulk component as the mesa area increases, resulting in bulk dark current dominating the overall leakage behavior for larger devices. From linear fitting, the bulk and surface dark current densities are extracted as 3.28 mA/cm^2^ and 5.51 μA/cm at −1 V, respectively. Notably, the surface leakage density is somewhat higher than reported values for other Ge-based PDs, which was mainly attributed to sidewall damage induced during the chlorine-based dry-etching process. The resulting sidewall roughness increases the density of surface states, thereby promoting minority carrier generation at the Ge surface, enhancing surface leakage, and consequently elevating the total dark current.

**Figure 4:**
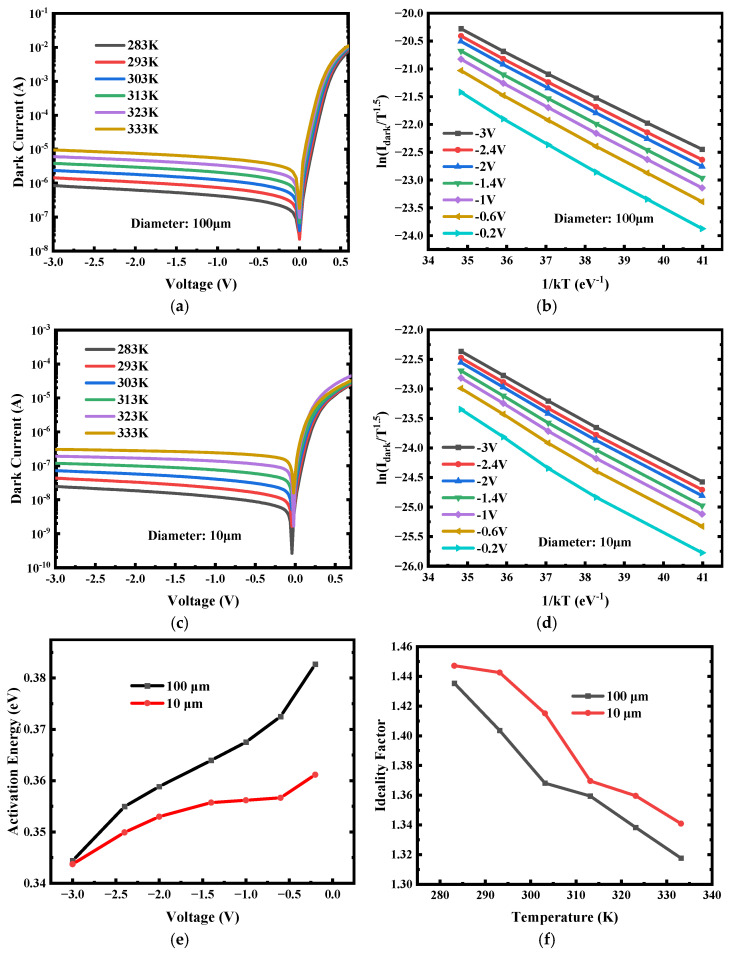
Temperature-dependent electrical characterization of the fabricated low-defect bulk-GeOI PD: (**a**,**c**) I–V curves measured at temperatures ranging form 283–333 K; (**b**,**d**) Arrhenius plot of ln(I_dark_) versus 1/kT for the extraction of activation energy; (**e**) Extracted activation energy (Ea) as a function of reverse bias voltage; and (**f**) extracted ideality factor as a function of reverse bias voltage.

While the leakage mechanisms in epitaxial GeOI PDs have been extensively studied [64,65,66], the dark current behavior of devices fabricated on bonded bulk-GeOI substrates remains largely unexplored. As discussed above, the surface and bulk contributions were decoupled by analyzing the dependence of dark current density on mesa diameter, with the surface component primarily associated with sidewall roughness and passivation quality—factors also commonly observed in epitaxial Ge devices. The bulk dark current, in contrast, arises from three fundamental mechanisms: (i) diffusion current, originating from thermally generated minority carriers in the P and N regions that diffuse to the depletion edge and are swept by the built-in electric field; (ii) recombination current, associated with carrier generation via defect-related Shockley–Read–Hall (SRH) [67,68] centers within or near the depletion region, where the electric field rapidly separates the generated carriers; and (iii) tunneling current, which becomes prominent under high doping or strong electric fields, encompassing both band-to-band and trap-assisted tunneling processes. Each mechanism exhibits a distinct temperature dependence, reflecting its underlying physical origin. To further elucidate the leakage behavior in bonded low-defect bulk-GeOI PDs, temperature-dependent dark current measurements were conducted, as illustrated in Figure 4a,c. Temperature dependent dark current was quantitatively described using the following Equation (2) [69]:

(2) $$I_{\mathrm{dark}}{=\mathrm{AT}}^{1.5}e^{-\frac{E_{a}}{\mathrm{kT}}}{(e}^{\frac{\mathrm{qV}}{2\mathrm{kT}}}-1)$$

where A is a pre-exponential constant, T is the absolute temperature, k is Boltzmann’s constant, and E_a_ represents the effective activation energy associated with the dominant leakage mechanism within the examined temperature and bias range. By plotting ln(I_dark_) versus 1/kT (Arrhenius plot, Figure 4b,d), E_a_ can be determined from the slope of the linear region [70,71]. In the measured temperature range, two distinct linear regimes are observed in the Arrhenius plots, corresponding to different dominant transport mechanisms. The lower-to-intermediate temperature regime reflects defect-assisted and diffusion-related processes, while at higher temperatures, intrinsic carrier excitation becomes dominant, leading to a larger apparent activation energy approaching the Ge bandgap energy scale. The activation energies presented in Figure 4e were extracted from the lower-to-intermediate temperature regime to specifically evaluate defect-related and diffusion-limited leakage mechanisms. Figure 4e presents the extracted activation energy as a function of reverse bias. Within 0 to −3 V, E_a_ exceeds 0.33 eV (i.e., >E_g_/2), indicating that the reverse dark current is dominated by diffusion-limited transport. As the reverse bias increases, E_a_ gradually approaches E_g_/2, suggesting an increasing contribution from SRH-mediated generation–recombination current. This interpretation is supported by comparing the activation energies of devices with different mesa diameters: the smaller 10 µm device exhibits a lower E_a_ than the 100 µm device, consistent with its larger perimeter-to-area ratio that amplifies the relative weight of surface-mediated SRH recombination. The dominance of diffusion current under low-to-moderate bias conditions reflects the high crystalline quality of the bonded bulk-Ge material, where defect-related recombination is minimal, and the leakage is primarily governed by intrinsic semiconductor properties. The temperature-dependent diffusion current [72] on fundamental material parameters can be described by Equation (3):

(3) $$I_{\mathrm{diff}}\propto \frac{n_{i}^{2}}{\tau \cdot N}$$

where n_i_ is the intrinsic carrier concentration (determined by E_g_ and T), τ denotes the minority carrier lifetime, and N represents the doping concentration in the respective region. The minority carrier lifetime τ is fundamentally governed by the density of recombination centers [73,74], such as material defects and impurities. A lower defect density corresponds to an extended τ, which enhances the diffusion current. The observed increase in diffusion current thus provides direct evidence of the high crystalline quality of the bonded bulk-Ge material, implying a reduced concentration of bulk recombination centers.

In addition, we investigated the forward-bias dark current and extracted the ideality factor [75], as shown in Figure 4f. The obtained ideality factor ranges from 1.3 to 1.46, indicating that the forward current arises from a combination of diffusion and generation–recombination mechanisms. With decreasing temperature, the contribution of the generation–recombination [76] component becomes increasingly dominant, due to the suppression of intrinsic diffusion current at lower temperatures, which accentuates the effect of surface leakage. The diffusion current density scales with n_i_^2^, which decreases exponentially with temperature as n_i_^2^∝T^3^exp (−Eg/kT). This exponential reduction dominates over other weaker temperature-dependent parameters such as mobility or diffusion coefficient, making the n_i_ dependence the principal mechanism. As temperature decreases, n_i_ drops exponentially, so the diffusion component is strongly reduced, and other leakage components such as depletion-region generation–recombination and surface-related leakage become relatively more dominant in the total dark current. The higher ideality factor observed for the 10 µm device compared to the 100 µm device further illustrates this point. This trend is consistent with the elevated surface leakage previously attributed to sidewall etching damage, highlighting the necessity for further process optimization to mitigate surface-related defects.

**Figure 5:**
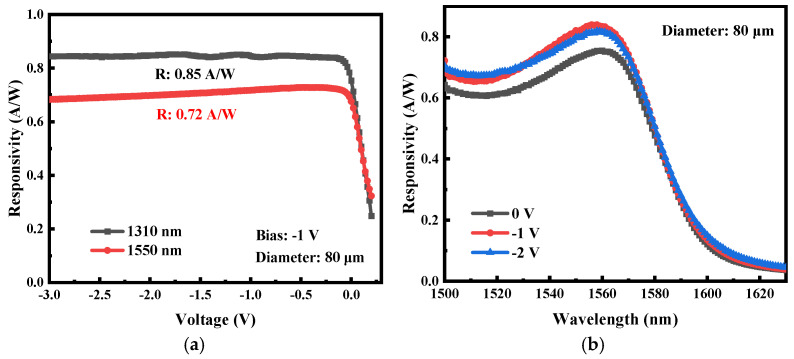
(**a**) The relationship graph between the response rate and voltage for a PD with an 80 μm diameter at 1310 nm and 1550 nm; (**b**) spectral response of 1550 nm–1630 nm of a low-defect bulk GeOI PD with 80 µm diameter.

The photoresponsivity of the fabricated low-defect bulk-GeOI PDs was characterized at 1310 nm and 1550 nm using a fiber-coupled laser source with an input power of 1 mW. The optical power at the fiber output was calibrated with a commercial power meter prior to device illumination. As shown in Figure 5a, the detector demonstrates high responsivity even at zero bias, reaching 0.75 A/W at 1310 nm and 0.68 A/W at 1550 nm. Near-saturation responsivity is achieved at a low reverse bias of −0.1 V, highlighting the device’s potential for ultra-low-power operation. Under a −1 V reverse bias, the responsivities further increase to 0.85 A/W (1310 nm) and 0.72 A/W (1550 nm). The corresponding external quantum efficiency (EQE) was calculated according to Equation (4):

(4) $$\mathrm{EQE}=\frac{R\cdot \mathrm{hc}}{q\lambda }\times 100\%$$

where R is the responsivity, h is Planck’s constant, c is the speed of light, and λ denotes the incident wavelength. The resulting external quantum efficiency (EQE) reaches 80.6% at 1310 nm and 57.7% at 1550 nm, surpassing the values reported for GOI detectors with comparable geometries [77], representing an 84% improvement specifically at 1550 nm. The enhanced performance can be primarily attributed to two factors: (i) the intrinsically low defect density of the bulk Ge layer significantly reduces the density of carrier recombination centers, enabling photogenerated carriers to efficiently traverse the intrinsic region and be collected at the electrodes; and (ii) lower unintentional doping concentration in the bulk-Ge intrinsic region, relative to epitaxially grown Ge, suppresses dopant-assisted carrier recombination and facilitates more efficient electron-hole separation under the applied electric field [78], thereby enhancing the overall quantum efficiency. Additionally, the spectral responsivity of the device was characterized over the wavelength range of 1500–1630 nm, as shown in Figure 5b. A pronounced responsivity peak is observed at 1550 nm, indicating enhanced optical absorption at this wavelength. This is mainly because the designed 800 nm-thick oxide layer functions as a resonant cavity, selectively enhancing the optical field near 1550 nm through constructive interference between the incident light and the reflection at the Ge/oxide interface. While the resonant cavity also enhances absorption efficiency, the overall responsivity is governed by both absorption and collection efficiency. Compared to other resonant-cavity-enhanced Ge devices, our responsivity is superior, confirming that the low defect density of bulk Ge plays a critical role. In addition, we also calculated the specific detectivity (D*) of the device. For our 80 μm device at −1 V, the specific detectivity reaches 1.43 × 10^10^ cm·Hz^1/2^·W^−1^ at 1550 nm and 1.69 × 10^10^ cm·Hz^1/2^·W^−1^ at 1310 nm, which represents a competitive level compared to other devices [79].

To further verify the enhanced optical absorption of our device at 1550 nm, its performance is benchmarked against previously reported vertically illuminated GeOI PDs. As summarized in Table 1, the present bulk GeOI detector employs a relatively thin intrinsic absorption layer of 800 nm, and achieves a high responsivity of 0.72 A/W at 1550 nm, corresponding to an external quantum efficiency (EQE) of 57.7%. This performance surpasses that of epitaxial GeOI devices with thicker intrinsic layers (~1.2 μm) [80] and is competitive even with some resonant-cavity-enhanced designs that incorporate distributed Bragg reflectors (DBRs) [81]. Moreover, a responsivity of 0.85 A/W is attained at 1310 nm. While our bulk current is not the lowest, the overall performance demonstrates the clear potential of the bulk Ge approach. Further suppression of sidewall leakage in future work will allow this bulk advantage to be more fully realized. These results demonstrate that efficient photodetection in the SWIR band can be realized with a thin absorber, thereby offering a new route to co-optimize speed and quantum efficiency [82,83,84].

The enhanced responsivity stems primarily from the superior crystal quality of the bulk Ge material. Compared with epitaxially grown Ge, bulk Ge exhibits an extremely low defect density (FWHM is only 72 arcsec), which greatly reduces the probability of photogenerated carriers being trapped by defect states and undergoing non-radiative recombination. As a result, the minority carrier lifetime is significantly extended [85], enabling efficient collection of carriers generated within the optical penetration depth even in a thin absorption layer. Concurrently, the buried oxide layer in the GOI platform provides optical reflection that enhances the round trip absorption of light, further boosting the EQE. This combined strategy of “material quality enhancement” and “optical field engineering” allows the detector to maintain a thin profile and high speed potential while delivering strong broadband responsivity. In summary, this work shows that employing high-quality bulk Ge as the absorber can effectively improve the absorption and conversion efficiency of Ge-based detectors at 1550 nm. The approach provides a feasible and CMOS-compatible route toward thin-layer, high-performance SWIR PDs.

**Table 1:** Performance Comparison of Vertically Incident Ge Detectors.

Year	Structure/Materials	Area (μm^2^)	I_dark_ (nA)	J_bulk_ (mA/cm^2^)	d_intrinsic_ (nm)	R(A/W)/EQE at 1310 nm	R(A/W)/EQE at 1550 nm	Ref.
2017	NIP/EPI GeOI (SiO_2_/Si_x_N_y_/SiO_2_)	π × 30^2^	1329	47	822	——	0.39/31.3%	[60]
2020	NIP/EPI GeOI (SiO_2_/Si_x_N_y_/SiO_2_)	π × 125^2^	280	0.46	350	——	0.28/22.4%	[77]
2021	NIP/EPI GeOI w/o TEOS	π × 5^2^	2.5	1.79	1200	0.7/66.4%	0.43/34.5%	[80]
2021	NIP/EPI GeOI w/TEOS	π × 5^2^	2.7	2.02	1200	0.9/85.3%	0.5/40%	[80]
2021	NIP/EPI GeOI (SiO_2_/Si_x_N_y_/SiO_2_)	π × 40^2^	40	0.37	700	——	0.29/23.2%	[86]
2024	NIP/EPI GeOI w/mirror	π × 5^2^	5.75	5.7	~1200 *	0.85/80.6%	0.67/53.7%	[87]
2026	PIN/Bulk GeOI	π × 40^2^	299	3.28	800	0.85/80.6%	0.72/57.7%	This work

* The data represent the estimated values calculated based on the figures provided in the literature.

**Figure 6:**
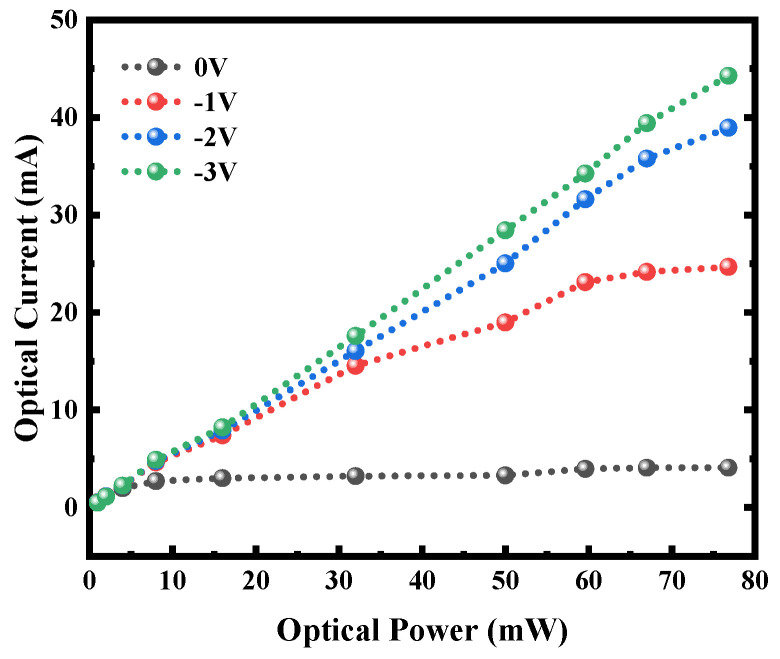
The saturation photocurrent curve of a low-defect Bulk-GeOI PD at 1550 nm.

We further evaluated the high-power characteristics of the low-defect Bulk-GeOI PD. Under intense optical illumination, the device shows an excellent photocurrent response, as depicted in Figure 6. When the incident optical power increases from 0 to nearly 80 mW, the output photocurrent reaches 45 mA at a reverse bias of −3 V. Notably, the photocurrent behavior differs markedly under different bias conditions: at zero bias, the photocurrent saturates rapidly with increasing optical power, whereas at −3 V, it maintains a linear dependence over a wide power range (up to ~60 mW), demonstrating a substantially extended linear operation window [87]. This behavior originates from the influence of bias voltage on carrier transport and collection efficiency. Under zero bias, carrier separation relies solely on the built-in electric field. At high illumination levels, the field can become screened or insufficient to extract all photogenerated carriers promptly, leading to enhanced recombination and early photocurrent saturation. In contrast, a −3 V reverse bias widens the depletion region and strengthens the electric field, which not only accelerates carrier drift but also effectively suppresses the recombination of photogenerated electron-hole pairs. As a result, even under high-injection conditions, carrier collection remains efficient, enabling a broader linear response range [88]. These results demonstrate that the detector offers favorable high-power handling capability and linear photoresponse under reverse bias, making it suitable for photoelectric applications requiring a high dynamic range.

## 4. Conclusions

We have demonstrated the fabrication and characterization of high-performance PDs based on low-defect bulk-GeOI PDs for high-resolution SWIR imaging. The devices were fabricated by direct wafer bonding of high-quality bulk Ge, controlled thinning to ~1300 nm with an 800 nm intrinsic layer, precise n-type and p-type doping, and Al_2_O_3_/Si_3_N_4_ bilayer sidewall passivation. This process yields a platform with high crystalline quality and well-defined junctions. Optical measurements show high responsivities of 0.85 A/W at 1310 nm and 0.72 A/W at 1550 nm, corresponding to external quantum efficiencies of 80.6% and 57.7%, respectively. The enhanced photon-to-carrier conversion efficiency is primarily attributed to the resonant cavity effect and efficient carrier collection. Electrically, the measured dark current remains at the nA level (e.g., 18 nA at −1 V for a 10 μm-diameter device). Further analysis of the dark current shows that this leakage is dominated by surface and sidewall defects introduced during mesa etching, rather than by bulk material imperfections. This observation underscores that the bulk-GeOI platform itself inherently provides a low-defect foundation, with minimal bulk contribution to dark current. Overall, our findings highlight that the high-quality bulk-GeOI platform can deliver strong optical performance at telecom wavelengths. Further optimization of sidewall passivation, etching processes, and device integration will be essential to simultaneously reduce dark current and enhance responsivity in SWIR PDs.

## Data Availability

The data presented in this study are available on request from the corresponding authors.
